# Solving the unsolved rare diseases in Europe

**DOI:** 10.1038/s41431-021-00924-8

**Published:** 2021-06-18

**Authors:** Holm Graessner, Birte Zurek, Alexander Hoischen, Sergi Beltran

**Affiliations:** 1 Institute of Medical Genetics and Applied Genomics, University of Tübingen, Tübingen, Germany; 2 Centre for Rare Diseases, University Hospital Tübingen, Tübingen, Germany; 3 Department of Human Genetics, Radboud University Medical Center, Nijmegen, the Netherlands; 4 Radboud Institute for Molecular Life Sciences, Nijmegen, The Netherlands; 5 Department of Internal Medicine and Radboud Center for Infectious Diseases (RCI), Radboud University Medical Center, Nijmegen, the Netherlands; 6 CNAG-CRG, Centre for Genomic Regulation (CRG), The Barcelona Institute of Science and Technology, Barcelona, Spain; 7 Universitat Pompeu Fabra (UPF), Barcelona, Spain; 8 Departament de Genètica, Microbiologia i Estadística, Facultat de Biologia, Universitat de Barcelona (UB), Barcelona, Spain

Getting a diagnosis can be a long and arduous process for a patient with a rare disease. Some patients take months, years, or even their entire lives to receive a proper diagnosis and occasionally corresponding therapy. Around 50 percent of patients with a rare disease remain undiagnosed even in advanced expert clinical settings where Whole Exome Sequencing (WES) is applied routinely as a diagnostic approach.

The European Reference Networks for Rare and Complex Diseases (ERNs) show that European collaboration can be a game changer when it comes to addressing rare disease needs. Solve-RD (Solving the Unsolved Rare Diseases)—a EU (Horizon 2020)-funded large-scale research project—leverages this ERN-related experience and outcome. For the first time in Europe hundreds of rare disease experts team up to actively share and jointly analyse existing data from unsolved rare disease patients. Involved in the project are data scientists and genomics experts as well as expert clinicians and geneticists from the ERNs.

The September edition of the journal features a series of six manuscripts describing the new collaborative approach, the structures established to warrant best exchange of data and expertise and the first results. These six studies mainly focus on one part of the Solve-RD’s large-scale data re-analysis approach, whereas results from the second main approach—the combined -omics approach – are expected towards the end of 2021.

Zurek et al. [1] describe the innovative clinical research environment of Solve-RD that introduces novel ways to organize data and expertise. Data scientists and genomics experts are organised in a Data Analysis Task Force, and expert clinicians and geneticists from each ERN are organized in Data Interpretation Task Forces. Partnering or associated ERN centres are contributing datasets from rare disease patients who remain undiagnosed after whole exome or genome sequencing. They are being re-analysed by the Data Analysis Task Force using e.g. EU-funded resources such as the RD-Connect Genome-Phenome Analysis Platform (https://platform.rd-connect.eu).

In an accompanying paper, Matalonga et al. [2]—describe the first results of the approach to solve rare diseases through programmatic reanalysis of genome-phenome data. It details the technological approaches that have been taken to enable a secure, fast and cost-effective automated re-analysis. Preliminary results speak to the value and functionality of the Solve-RD data re-analysis—255 previously unsolved cases from ~4500 families have already been newly diagnosed.

Four case reports from the involved core ERNs (ERN-ITHACA, ERN-RND, ERN-Euro NMD, ERN-GENTURIS) showcase the added value of the collaborative Solve-RD re-analysis approach. de Boer et al. [3]—belonging to ERN ITHACA (Intellectual disability, TeleHealth, Autism and Congenital Anomalies)—describes a male proband with a complex neurodevelopmental disorder for which the re-analysis found a disease causing variant in MT-TL1. Because of the atypical presentation, a mitochondrial disorder had not been considered clinically and therefore the mitochondrial DNA had not been analysed prior to Solve-RD. In retrospect, several features are seen that fit the spectrum described for MT-TL1. Töpf et al. [4]—on behalf of ERN-Euro NMD (neuromuscular diseases) present the diagnostic journey of a patient with cerebellar hypoplasia and spinal muscular atrophy (PCH1) and congenital bone fractures, that remained undiagnosed after exome sequencing. Re-analysis of the exome data by inclusion in the Solve-RD project resulted in the identification of a homozygous stop-gain variant in *TRIP4*, previously reported as disease causing. Thus, a novel genetic form of PCH1 could be identified, further strengthening the link of this characteristic phenotype with altered RNA metabolism. te Paske et al. [5]—representing ERN-GENTURIS (rare genetic tumour risk syndromes)—highlight mosaicism as a potential—and understudied—mechanism in the aetiology of diffuse gastric cancer. The joint re-analysis of exome data of unresolved hereditary gastrointestinal cancer patients revealed a pathogenic *PIK3CA* missense variant, c.3140 A > G p.(His1047Arg), in a 25-year-old patient diagnosed with diffuse gastric cancer. The variant, located in a known cancer-related somatic hotspot, was present at a low variant allele frequency in leucocyte-derived DNA. Somatic variants in *PIK3CA* are usually associated with overgrowth, a phenotype that was not observed in this patient. Schüle et al. [6]—belonging to ERN-RND (rare neurological diseases) describe three cases which have been solved by the Solve-RD re-analysis. These cases have not been solved earlier because (a) the respective genetic variant has been included in the ClinVar database after the initial genetic workup (example ITPR1 gene); (b) exon-intron boundaries commonly covered by WES allowed to find a non-coding variation causing RND (example *POLR3A*) and (c) initial research centred on disease-specific panels (example *EXOSC3* associated disease). The use of human phenotype ontology based on phenotypes rather than diagnostic categories as well as consideration of variant specific rather than gene-specific phenotypes enabled detection of pathogenic variants.

These case reports highlight that Solve-RD is having a positive impact on the diagnostic journey of individual patients and families. From a societal perspective, Solve-RD proves one main point: Collaborative European research is an ideal instrument to mobilise and organise the required interdisciplinary clinical and scientific resources to tackle *diagnosis of rare diseases*, a Pan-European and global healthcare problem, in order to impact how healthcare is being provided on national and local scale.

This series of Solve-RD papers is in memory of Prof. Gert-Jan van Ommen.
